# A new species of *Ceraeochrysa* Adams (Neuroptera, Chrysopidae), with a key to the species from Mexico

**DOI:** 10.3897/zookeys.888.39064

**Published:** 2019-11-11

**Authors:** Rodolfo J. Cancino-López, Atilano Contreras-Ramos

**Affiliations:** 1 Posgrado en Ciencias Biológicas, sede Instituto de Biología-UNAM, Cd. Universitaria, 04510 Ciudad de México, Mexico Posgrado en Ciencias Biológicas, sede Instituto de Biología-UNAM México Mexico; 2 Instituto de Biología-UNAM, Departamento de Zoología, Cd. Universitaria, 04510 Ciudad de México, Mexico Instituto de Biología-UNAM México Mexico

**Keywords:** Central American Volcanic Arc, Green lacewings, taxonomy, Volcán Tacaná

## Abstract

The genus *Ceraeochrysa* Adams is widely distributed in the New World, from southeastern Canada to Argentina, with 15 out of 61 previously known species recorded in Mexico. In this paper, *Ceraeochrysa tacanensis***sp. nov.** is described and illustrated from Volcán Tacaná, Chiapas, and an identification key to *Ceraeochrysa* species present in Mexico is provided. The new species is similar to others with swollen and darkened posterior branches of the cubital vein, and it can be separated from these other species by an elongate gonapsis extending from the base of the gonosaccus; the gonapsis is slightly upturned, terminating in a rounded apex with dorsal microteeth. Females of the new species have non-distinctive genitalia morphology. However, they can be associated with males of the species by body color pattern, synchrony, and sympatry.

## Introduction

The Neotropical green lacewing genus *Ceraeochrysa* (Neuroptera, Chrysopidae) was separated from *Chrysopa* by Adams (1982), who based his definition of the genus on male genitalic characters and recognized 24 species. Further studies added several species to this genus (Brooks and Barnard 1990; Penny 1997, 1998, 2002; Tauber et al. 2000; Freitas and Penny 2001; Tauber and De León 2001). *Ceraeochrysa* is the second most species-rich chrysopid genus in the New World after *Leucochrysa* McLachlan, comprising 61 valid species (Sosa and Freitas 2010, 2011; Tauber and Flint 2010; Tauber and Garland 2014).

This genus is distributed from southeastern Canada to Argentina, and its greatest species richness and abundance is in the tropics (Adams 1982; Brooks and Barnard 1990; Freitas et al. 2009; Tauber et al. 2000; Sosa and Freitas 2010). Currently, countries having the highest species richness of *Ceraeochrysa* include Brazil (33 species), Costa Rica (23), Mexico (15), Panama (14), and Venezuela (12) (Freitas et al. 2009; Sosa and Freitas 2010; Oswald 2018; Martins and Machado 2019). Species of this genus have been reported from dry and open forests and various agroecosystems (Tauber et al. 2000; Freitas et al. 2009). Their larvae are trash-bearers and feed on soft-bodied arthropods such as aphids, diaspidids, thrips, aleyrodids, psyllids, and neonatal larvae of Lepidoptera, which makes them potentially useful for biological control (Tauber et al. 2000; Freitas 2001; Penny 2002; Freitas et al. 2009).

There have been few studies of the Chrysopidae of Mexico, and knowledge of this group is fragmented. The aim of this paper is to describe and illustrate a new species of the genus *Ceraeochrysa* as part of a survey of the lacewings of the Tacaná Volcano, Chiapas across an altitudinal gradient. Also, a key to males of the species of this genus known from Mexico is included, excluding *C. indicata* (Navás) and *C. lateralis* (Guérin-Méneville) for which males are unknown. Due to their potential importance in the biological control of agricultural pests, there is an established need to better describe the green lacewing fauna of Mexico.

## Materials and methods

The material examined was obtained during monthly samplings (February 2018–January 2019) in the Tacaná Volcano Biosphere Reserve, Chiapas state, Mexico. Specimens were captured at lights traps and with aerial net on vegetation, kept alive in plastic screw cap vials, then they were pinned as they died, or after being killed by freezing. For dissection of genitalia, the abdomen was cut between the 6^th^ and 7^th^ segments and the apical segments were removed and cleared with solution of 10% potassium hydroxide (KOH) for 15 min at 80 °C in a water bath. The cleared genitalia were stained using Clorazol Black E and then placed in microvials with glycerin. Observations were done under a Discovery V8 Zeiss dissecting microscope. Serial images from different layers were taken with a Zeiss Axio Zoom V16 microscope fitted with an AxioCam MRc5 digital camera and stacked using Zen 2012 (Blue edition). Head width was measured as the distance between the outer margins of the eyes, dorsally. Wing length was measured from the joint region to the apex (Sosa and Freitas 2010). The holotype and allotype, both dissected, are deposited at the Colección Nacional de Insectos (**CNIN**) of the Instituto de Biología, UNAM, Mexico City; paratypes will be deposited at CNIN, the Colección de Insectos asociados a plantas cultivadas en la Frontera Sur (**ECO-TAP-E**) and the National Museum of Natural History, Smithsonian Institution (**NMNH**), Washington, DC. The key was constructed based on Freitas et al. (2009).

## Taxonomy

### Ceraeochrysa tacanensis

Taxon classificationAnimaliaNeuropteraChrysopidae

Cancino-López & Contreras-Ramos sp. nov.

271B16C8-0E7C-5BBA-9089-B5B1A34872F5

http://zoobank.org/6B20810F-BA84-4838-AF7B-9AD9837497B4

Figures 1 , 2 , 3

#### Material examined

**(20 males, 11 females). *Holotype* (male)**: MEXICO: Chiapas, Cacahoatán, Ej[ido] Benito Juárez El Plan, 15°05'27.18"N, 92°08'51.06"W, 1479 m, 17.ii.2018, Cancino-López & Luna-Luna, light trap [genitalia dissected] (CNIN). ***Allotype***: MEXICO: Chiapas, Unión Juárez, Cantón Chiquihuites, 15°05'46.26"N, 92°05'56.46"W, 2072 m, 16.iv.2018, Cancino-López & Luna-Luna, light trap [genitalia dissected] (CNIN). ***Paratypes***: MEXICO: Chiapas, Cacahoatán, Ej[ido] Benito Juárez El Plan, 15°05'27.18"N, 92°08'51.06"W, 1479 m, 17.ii.2018, Cancino-López & Luna-Luna, light trap, 1 male, 1 female [genitalia dissected] (CNIN); same data but, 15°05'13.02"N, 92°08'55.2"W, 1430 m, 16.iii.2018, 2 males [one with genitalia dissected] (CNIN); same data but, 15°05'53.28"N, 92°08'29.88"W, 1705 m, 16.iii.2018, Cancino-López, 1 female, entomological net (CNIN); same data but, 15°05'36.48"N, 92°08'43.92"W, 1553 m, 12.viii.2018, 2 males (CNIN); same data but, 15°05'37.74"N, 92°08'43.26"W, 1572 m, 1 male (CNIN); same data but, 15°05'27.18"N, 92°08'51.06"W, 1479 m, 20.ix.2018, Cancino-López & Luna-Luna, 1 female, light trap (CNIN); same data but, 15°05'41.94"N, 92°08'41.52"W, 1577 m, 06.x.2018, Cancino-López, 1 female, entomological net (NMNH); same data but, 15°05'34.98"N, 92°08'45.42"W, 1541 m, 07.xi.2018, 1 female (NMNH); same data but, 15°05'40.98"N, 92°08'40.8"W, 1567 m, 08.xii.2018, 2 males (CNIN); same data but, 15°05'36.54"N, 92°08'43.8"W, 1549 m, 1 male (CNIN); same data but, 15°05'37.44"N, 92°08'43.68"W, 1564 m, 08.i.2019, 1 male (NMNH); same data but, 15°05'35.22"N, 92°08'44.76"W, 1533 m, 1 male (NMNH); same data but, 15°05'45.66"N, 92°08'40.5"W, 1582 m, 10.i.2019, 1 male, 1 female (ECO-TAP-E). MEXICO: Chiapas, Unión Juárez, Cantón Chiquihuites, 15°05'54.42"N, 92°05'57.96"W, 2157 m, 19.ii.2018, Cancino-López & Luna-Luna, 1 male [genitalia dissected], light trap (CNIN); same data but, 15°05'46.26"N, 92°05'56.46"W, 2076 m, 16.iv.2018, 1 male (CNIN); same data but, 15°05'43.74"N, 92°05'57.6"W, 2060 m, 14.v.2018, 3 males (CNIN); same data but, 15°05'43.79"N, 92°05'57.6"W, 2081 m, 10.ix.2018, 1 male, 1 female (NMNH); same data but, 15°05'43.79"N, 92°05'57.6"W, 08.x.2018, 1 male (CNIN); same data but, 15°06'9.06"N, 92°06'18.42"W, 2430 m, 19.xi.2018, Cancino-López, 1 male, entomological net (NMNH); same data but, Almaraz-Hernández, 1 female (NMNH); same data but, 15°05'43.79"N, 92°05'57.6"W, 2081 m, 14.i.2019, Cancino-López & Luna-Luna, 1 male, light trap (CNIN).

#### Diagnosis.

**Figure 1.**
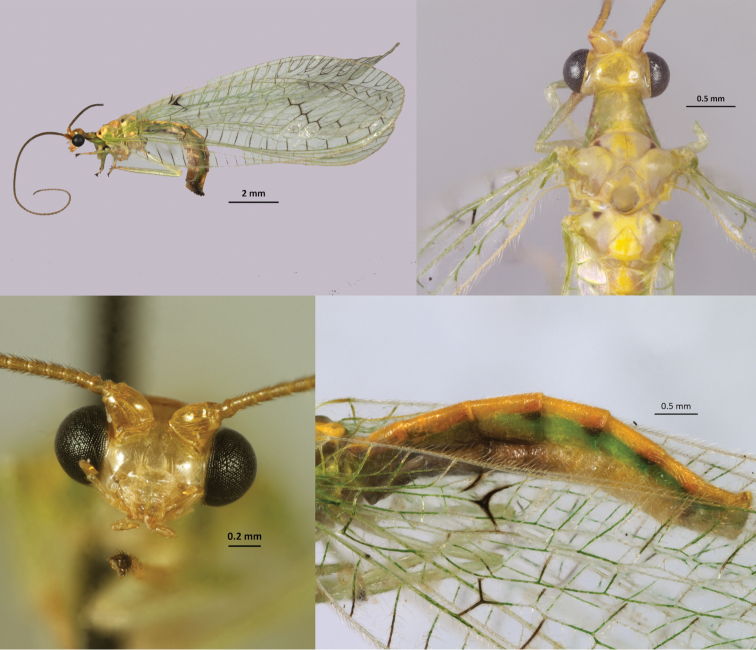
*Ceraeochrysa tacanensis* sp. nov. **A** habitus, lateral **B** head and thorax, dorsal **C** head, frontal **D** abdomen, lateral.

This species has marks on the pronotum (a discontinuous red lateral stripe) and on the meso- and metanota (two anterior reddish black spots on each) (Fig. 1B) and on the abdominal tergites (orange to dark-brown lateral elongate marks) (Fig. 1D); forewing has the posterior branches of the cubital vein swollen, darkened and edged with dark on the membrane; last tarsal segments are darkened (Fig. 1A). The gonosaccus basally bears gonosetae (Fig. 3A); the arcessus is very long, narrow, straight, with curved apical point (Fig. 3B); the gonapsis is elongate, its basal section extends internally from the base of gonosaccus and is slightly upturned, terminating anteriorly in a smoothly rounded apex (Fig. 3E), the distal section extends externally and terminates dorsally with microteeth (Fig. 3D); a membranous sac between apices of gonapsis and sternite 9 bears a field of well-developed gonocristae (Fig. 3A).

#### Description.

**Measurements, mean (range) (*n* = 20). Male.** Head: width 1.3 mm (1.2–1.4 mm). Pronotum: length 0.85 mm (0.7–1 mm), width 0.6 mm (0.4–0.8 mm). Forewing: length 11.7 mm (10–13.4 mm); 4–6 inner and 5–7 outer gradate veins. Hindwing: length 10.2 mm (8.8–11.6 mm); 3–5 inner and 4–6 outer gradate veins. **Female (*n* = 11).** Head: width 1.2 mm (1.1–1.3 mm). Pronotum: length 10 mm (0.9–1.1 mm), width 0.95 mm (0.9–1 mm). Forewing: length 12.4 mm (11.9–12.9 mm); 5–6 inner and 7 outer gradate veins. Hindwing: length 13.5 mm (10.2–11.9 mm); five or six inner and six or seven outer gradate veins.

***Head*.** Front mainly pale (rarely with one brown, irregular transverse-stripe), vertex, clypeus, labrum, gena, maxillary, and labial palpi pale (Fig. 1C). Scape pale with lateral red stripe and pedicel pale with posterior-lateral red spot; flagellum pale, with 85–90 flagellomeres (*n* = 31).

***Thorax*.** Pronotum greenish with a discontinuous red lateral stripe on each side and a medial, longitudinal yellow band; meso- and metanota greenish, each with a medial, longitudinal yellow band and two anterior reddish-black spots (Fig. 1A), and sometimes with two posterior red or orange spots; pleura pale green. Legs: pale green with yellow tarsi, except one or two dark-brown apical tarsomeres (Fig. 1C). Forewings: venation mostly green, but some crossveins dark; dark markings at apex of 1A, posterior cubitus, and Cua-Cup crossveins form a distinct chevron-shaped mark (Fig. 1D); four to six inner and five to seven outer gradate veins. Hindwing: venation green, with apical section of radius dark; three to five inner and four to six outer gradate veins, all green.

***Abdomen*.** Green, with dorsal, longitudinal yellow band; tergites with orange to dark-brown lateral elongate marks at posterior margin (Fig. 1D). Male apodeme slightly sclerotized and thin, without ventral lobe (Fig. 3A).

***Male genitalia*.** Gonarcus thick with wide and elongate lateral plates (Fig. 3C); entoprocessus elongate, with evenly tapering tips (Fig. 3B); gonocornus lacking. Arcessus very long, narrow, straight, with downward curved apical point (Fig. 3B). Gonosaccus basally with gonosetae (Fig. 3A). Gonapsis elongate, extending from base of gonosaccus, slightly upturned, terminating internally in a smoothly rounded apex (Fig. 3E), with sclerotized microteeth on dorsal side (Fig. 3D); membranous sac between apices of gonapsis and sternite 9 with a field of well-developed gonocristae (Fig. 3A).

**Female.** Similar to holotype. *Female genitalia*. Female subgenitalia as wide as long, with rounded apex and narrow medial notch (Fig. 3G); spermatheca well sclerotized, with vela broad basally and strongly arched apically; spermathecal duct slightly sinuous before entering oviduct; ventral impression conspicuous (Fig. 3F).

#### Variation.

**Figure 2.**
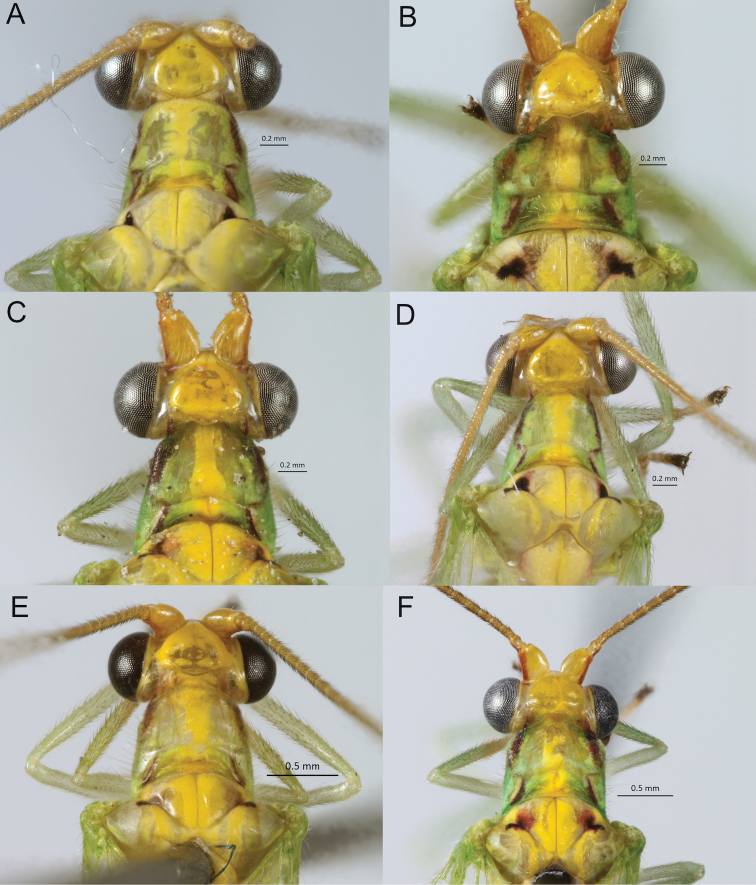
*Ceraeochrysa tacanensis* sp. nov., stripe variation on pronotum **A** discontinuous **B** interrupted **C** thickened **D** narrow **E** pale red **F** dark red.

Lateral stripes of pronotum are variable, for instance whether they are continuous or interrupted (Fig. 2A, B), thickened or narrow (Fig. 2C, D), pale or dark red (Fig. 2E, F); also, dorsolateral marks of the abdomen are generally orange, but may be reddish brown.

**Figure 3.**
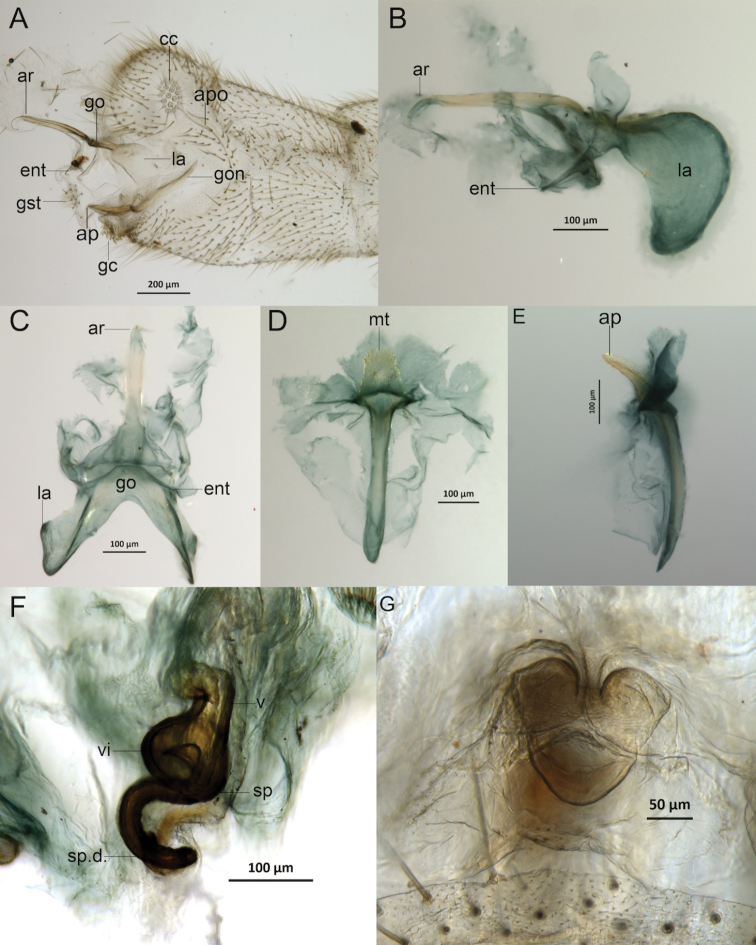
*Ceraeochrysa tacanensis* sp. nov. genitalia: **A** male terminalia, lateral **B** gonarcal complex, lateral **C** gonarcal complex, dorsal **D** gonapsis, dorsal **E** gonapsis, lateral **F** spermathecal complex, dorsolateral **G** female subgenitalia, frontal. Abbreviations: **ap**, apex of gonapsis; **apo**, male apodeme; **ar**, arcessus; **cc**, callus cerci; **ent**, entoprocessus; **gc**, gonocristae; **go**, gonarcus; **gon**, gonapsis; **gst**, gonosetae; **la**, lateral arms; **mt**, microteeth on gonapsis; **sp**, spermatheca; **sp.d.**, spermathecal duct; **v**, vela; **vi**, ventral impression.

#### Etymology.

This species is named after the Tacaná Volcano, located in the state of Chiapas, Mexico, where the specimens were collected.

#### Ecology.

This species is presently known from cloud forest (1,430–1,705 m a.s.l.) and mixed oak-cloud forest (2,060–2,430 m a.s.l), and with similar collecting techniques and collecting effort, it was not found at lower (661–1,393 m a.s.l.) or higher (2,884–3,246 m a.s.l) elevation collecting sites. Specimens were found on *Alinus* sp., *Quercus* sp., and *Saurauia* sp., and were collected from February through May, August through December 2018, and January 2019.

## Discussion

*Ceraeochrysa tacanensis* sp. nov. shares the posterior branches of the cubital vein swollen and dark, V-shaped marking with *C. angulata* (Navás), *C. angusta* Freitas & Penny, *C. digitata* Freitas & Penny, *C. elegans* Penny, *C. nigripedis* Penny, and *C. tauberae* Penny. Also, an elongate arcessus is shared with these species (except *C. angulata* and *C. digitata*), plus *C. bitacornua* Freitas & Penny. The new species differs from the former species because it has a discontinuos stripe on the pronotum, while the rest have spots (*C. angulata*, *C. angusta*, *C. elegans*, *C. nigripedis*, and *C. tauberae*) or a continuous stripe (*C. bitacornua* and *C. digitata*). Another species with a discontinous stripe on the pronotum is *C. pittieri* Sosa & Freitas (Sosa and Freitas 2010: figs 4, 5), however, this species does not share other traits as explained above. In addition, *C. tacanensis* sp. nov. shares marks on the abdominal tergites with *C. elegans*, although the tarsal segments are darkened apically in the new species, as in *C. nigripedis*. Regarding genitalia, the new species is most similar to *C. nigripedis*, sharing a simple dorsal apodeme, an elongate gonapsis, and the shape of the gonarcal complex. However, the new species has a gonosaccus with gonosetae and a membranous sac with gonocristae between apex of gonapsis and sternite 9, similar to *C. elegans*. The sclerotized microteeth extended on the dorsal side of the gonapsis apex may be a unique trait of the new species (also present in the unrelated *C. sanchezi*), while *C. elegans* has microteeth restricted to the apex.

### Key to species of *Ceraeochrysa* of Mexico (Modified from Freitas et al. 2009)

**Table:** 

1	Pronotum with one or more pairs of lateral spots, or thin, sub-medial stripes	**2**
–	Pronotum with red or brown lateral stripes or no stripes	**3**
2	Last two tarsal segments of legs black; lateral surface of antennal scape red; abdominal tergites with orange spots	***Ceraeochrysa tacanensis* Cancino-López & Contreras, sp. nov.**
–	Tarsal segments of legs pale; lateral surface of antennal scape dark; abdominal tergites with red bands	***C. elegans* Penny**
3	Area of vertex behind antennal bases entirely red	***C. smithi* (Navás)**
–	Area of vertex behind antennal bases pale	**4**
4	Basal flagellar segments pale	**5**
–	Basal flagellar segments dark	**9**
5	Maxillary palpi pale, with dark marks	***C. cubana* (Hagen)**
–	Maxillary palpi pale, without dark marks	**6**
6	Antennal scape with two stripes	***C. arioles* (Banks)**
–	Antennal scape with one stripe	**7**
7	Antennal scape with lateral stripe	***C. valida* (Banks)**
–	Antennal scape with dorsal stripe	**8**
8	Mesonotum with dark marks; male dorsal apodeme with long ventral branch, basally attached; arcessus as broad as long; gonapsis thick and short	***C. cornuta* (Navás)**
–	Mesonotum unmarked; male dorsal apodeme with recurved ventral branch basally attached; arcessus broad; gonapsis long, slender, apically upturned	***C. cincta* (Schneider)**
9	Antennal scape with lateral or dorsolateral stripe/spot	**10**
–	Antennal scape with dorsal stripe	***C. claveri* (Navás)**
10	Genae dark to partially dark	**11**
–	Genae pale yellow to pale brown	**13**
11	Apex of male ectoproct rounded, with simple, thin setae	***C. derospogon* Freitas and Penny**
–	Apex of male ectoproct pointed, with chalazae (thick-based setae)	**12**
12	Male tergite 9 + ectoproct deeply divided; gonosaccus with field of gonocristae; sternite 8 + 9 quadrate with one long chalazate seta at each lateral corner; ventral fork of dorsal apodeme not projected caudally beyond ectoproct	***C. berlandi* (Navás)**
–	Male tergite 9 + ectoproct not deeply divided; gonosaccus lacking field of gonocristae; sternite 8 + 9 rounded with chalazate setae throughout; ventral fork of dorsal apodeme projected ventrocaudally well beyond ectoproct	***C. effusa* (Navás)**
13	Arcessus membranous basally with a pair of hooks and two inflated lobes, apex with a medial hook and pair of lateral, decurved and medially curved sclerotized lobes	***C. everes* (Banks)**
–	Arcessus not membranous basally, of triangular-shape; apex with medial decurved point	***C. sanchezi* (Navás)**

## Supplementary Material

XML Treatment for Ceraeochrysa tacanensis
